# Optimization of time to initial vancomycin target trough improves clinical outcomes

**DOI:** 10.1186/s40064-015-1146-9

**Published:** 2015-07-19

**Authors:** Anthony P Cardile, Christopher Tan, Michael B Lustik, Amy N Stratton, Cristian S Madar, Jun Elegino, Günther Hsue

**Affiliations:** Department of Medicine, Tripler Army Medical Center, 1 Jarrett White Roadm, Honolulu, HI 96859 USA; Department of Pharmacy, Tripler Army Medical Center, 1 Jarrett White Roadm, Honolulu, HI 96859 USA; Department of Clinical Investigation, Tripler Army Medical Center, 1 Jarrett White Roadm, Honolulu, HI 96859 USA; Department of Infectious Diseases, Tripler Army Medical Center, 1 Jarrett White Roadm, Honolulu, HI 96859 USA

**Keywords:** Vancomycin, MRSA, TDM, Trough

## Abstract

**Background:**

Outcomes data for the efficacy of interventions designed to decrease the time to initial target vancomycin troughs are sparse.

**Objective:**

A vancomycin therapeutic drug monitoring (TDM) program was initiated to reduce the time to initial target troughs and to examine the impact on clinical outcomes.

**Methods:**

Single-center, pre- and post-intervention observational study in a 250 bed teaching facility. Adult inpatients treated with physician-guided, vancomycin therapy (historical control, CTRL) were compared to high trough, pharmacist-guided vancomycin therapy (TDM). Nephrotoxicity analyses were conducted to the ensure safety of the TDM. Clinical outcome analysis was limited to patients with normal renal function and culture-confirmed gram positive infections and a pre-defined MRSA subset.

**Results:**

340 patients met initial inclusion criteria for the nephrotoxicity analysis (TDM, n = 173; CTRL, n = 167). Acute kidney injury occurrence was similar between the CTRL (n = 20) and TDM (n = 23) groups (p = 0.7). Further exclusions yielded 145 patients with gram positive infections for clinical outcomes evaluation (TDM, n = 66; CTRL, n = 75). The time to initial target trough was shorter in the TDM group (3 vs. 5 days, p < 0.001). Patients in the TDM group discharged from the hospital more rapidly, 7 vs. 14 days (Hazards Ratio (HR), 1.41; 95% Confidence Interval [CI] 1.08–1.83; p = 0.01), reached clinical stability faster, 4 vs. 8 days (HR, 1.51; 95% CI 1.08–2.11; p = 0.02), and had shorter courses of vancomycin, 4 vs. 7 days (HR, 1.5; 95% CI 1.15–1.95; p = 0.003). In the MRSA infection subset (TDM, n = 36; CTRL, n = 35), patients in the TDM group discharged from the hospital more rapidly, 7 vs. 16 days (HR, 1.89; 95% CI 1.08–3.3; p = 0.03), reached clinical stability faster, 4 vs. 6 days (HR, 2.69; 95% CI 1.27–5.7; p = 0.01), and had shorter courses of vancomycin, 5 vs. 8 days (HR, 2.52; 95% CI 1.38–4.6; p = 0.003). Attaining initial target troughs in <5 days versus ≥5 days was associated with improved clinical outcomes. All cause in-hospital mortality, and vancomycin treatment failure occurred at comparable rates between groups.

**Conclusions:**

Interventions designed to decrease the time to reach initial target vancomycin troughs can improve clinical outcomes in gram positive infections, and in particular MRSA infections.

**Electronic supplementary material:**

The online version of this article (doi:10.1186/s40064-015-1146-9) contains supplementary material, which is available to authorized users.

## Background

Methicillin-resistant *S. aureus* (MRSA) infections are a significant problem in both healthcare and community settings. Healthcare-associated methicillin-resistant *S. aureus* is frequently associated with invasive disease, such as skin and soft tissue infection, bloodstream infection (BSI), and pneumonia. In contrast, community-associated methicillin-resistant *S. aureus* is classically associated with skin and soft tissue infections, and necrotizing pneumonia in young, otherwise healthy persons. Vancomycin is most commonly utilized in the treatment of proven or suspected MRSA infections. Over time, there has been an increase in vancomycin resistance with subsequent treatment failure in MRSA infections. In addition, there have been concerns about the tissue penetration of vancomycin to sites of infection (most notably the lung) (Rybak et al. 2009). As a result, current dosing guidelines have advocated significantly higher doses of vancomycin than in the past (Rybak et al. 2009).

Vancomycin dosing and drug monitoring has been the subject of deliberation over the years (Rybak et al. 2009). Vancomycin drug level monitoring has been advocated to lessen the potential for nephrotoxicity and to achieve therapeutic concentrations (Rybak et al. 2009). However, opponents of monitoring cite the lack of evidence relating to meaningful clinical outcomes, and uncertainties about the role of vancomycin in nephrotoxicity (Rybak et al. 2009). Others highlight the increased cost and personnel time associated with monitoring (Rybak et al. 2009).

To address dosing and therapeutic monitoring of vancomycin in adult patients, a consensus statement was released in January 2009 advocating higher vancomycin doses (Rybak et al. 2009). There is significant concern that higher vancomycin doses and troughs carry an increased risk for nephrotoxicity and this has been suggested by recent studies (Hazlewood et al. 2010). Vancomycin-associated nephrotoxicity risk is higher in critically ill patients, patients receiving concomitant nephrotoxins, and those with chronic kidney disease (Hazlewood et al. 2010; Vandecasteele and De Vriese 2010). Some suggest that increased nephrotoxicity rates attributed to aggressive vancomycin dosing in recent studies may be related to selection bias as such patients are more likely to receive concomitant nephrotoxins and have other risk factors for nephrotoxicity (Hazlewood et al. 2010). However, a recent systematic review and meta analysis found that vancomycin-associated nephrotoxicity was significantly higher with vancomycin levels ≥15 mg/L (Steinmetz et al. 2015). With nephrotoxicity in mind, there has been a large body of research to investigate the optimal manner to safely achieve target troughs to include use of vancomycin dosing nomograms, pharmacokinetic modeling software, computerized prescriber-order-entry systems, and pharmacist managed therapeutic drug monitoring (TDM) programs (Aubron et al. 2011; Golenia et al. 2013; Kullar et al. 2012; Leu et al. 2012; Li et al. 2012; McCluggage et al. 2010; Minne et al. 2012; Morrison et al. 2012; Nunn et al. 2011; Patanwala et al. 2009; Pea et al. 2009; Revilla et al. 2010; Swartling et al. 2012; Traugott et al. 2011; Truong et al. 2012). TDMs have been shown to increase dosing efficiency and accuracy, reduce drug toxicity, and decrease hospitalization costs (Bond and Raehl 2005; Corallo et al. 2011; Fernández de Gatta et al. 1996; Iwamoto et al. 2003; Welty and Copa 1994). Most TDM studies published focus on whether or not the target level was reached and the efficiency of trough monitoring (Golenia et al. 2013; Leu et al. 2012; Minne et al. 2012; Morrison et al. 2012). The time to reach a therapeutic vancomycin trough is beginning to be recognized as an important factor (Li et al. 2012). However, few studies have examined this variable, and none to our knowledge have conducted a study to look at this parameter specifically (Aubron et al. 2011; Clemens et al. 2011; Gawronski et al. 2013; McCluggage et al. 2010). There has been no suggested time-frame at which a goal trough should be reached, though the consensus guidelines recommend a loading dose of 25–30 mg/kg to facilitate rapid attainment (Rybak et al. 2009). The concept of reducing the time to therapeutic trough is enticing as it parallels evidence that receipt of early, appropriate antibiotic therapy in severe infections is likely to improve clinical outcomes (Kumar et al. 2006). The time to reach target trough is discussed briefly in the 2009 consensus guidelines in relation to the study by Jeffres et al. (2006) and note that this may be an important predictor of outcome (Rybak et al. 2009). Thus, it was the purpose of the current study to institute a vancomycin TDM with the goal of reaching target vancomycin troughs faster, ensuring there is no increased risk of nephrotoxicity, and evaluating the subsequent impact on clinical outcomes for all empirically treated gram positive infections and culture proven MRSA infections.

## Methods

### Study design and patient population

**Figure 1:**
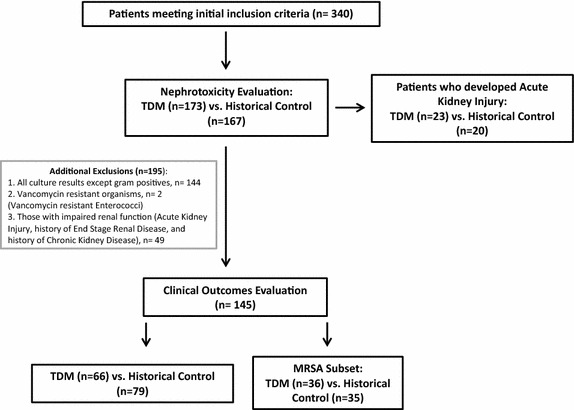
Study design.

This was a pre- and post-intervention observational study at Tripler Army Medical Center (Honolulu, HI, USA), a 250 bed teaching facility. The study protocol was approved by the Human Use Committee. Investigators adhered to the policies for protection of human subjects as prescribed in 45 CFR 46. Patients treated with vancomycin from July 2007–March 2008 (historical control, CTRL) were compared to those treated with vancomycin therapy via a pharmacist run, continuously active vancomycin therapeutic drug monitoring (TDM) program from July 2009–March 2010 (TDM). Those who received fewer than four doses of vancomycin and/or no troughs were drawn were excluded by initial automated data search leaving 340 patients for analysis. The development of acute kidney injury (AKI) and the characteristics of these patients were compared between the groups for all patients in the initial dataset for nephrotoxicity evaluation to determine the safety of the TDM intervention (Figure 1). Those with gram negative or no positive culture result (n = 144), those with vancomycin resistant organisms (n = 2), and those with chronic kidney disease (CKD) stages III/IV/V (n = 49) were excluded (Figure 1). We also examined a predefined subset of culture confirmed MRSA infections only (Figure 1).

### Description of the Intervention

To reduce the time to target trough, vancomycin therapy was guided by a standardized, pharmacist managed, TDM program. The vancomycin TDM was implemented hospital-wide and was active continuously. A cause and effect approach, adapted from business models, such as the Ishikawa diagram was utilized to identify systemic problems that impaired efficient and accurate dosing of vancomycin. Multidisciplinary interventions were then implemented to improve vancomycin dosing practices (Crowley et al. 2007). The critical vancomycin level was changed from 10 to 50 mg/L to avoid doses being inappropriately held by nursing staff. In addition “batching” of the troughs with daily morning lab draws was eliminated with emphasis on recording the actual “collected time” rather than the time the sample was run. This problem was further avoided by pharmacists scheduling the trough levels after “batched” morning laboratory specimens were collected. The goal to avoid batching was also based on the concept of attaining real-time test results to guide therapy, which has the potential to improve clinical outcomes (Barenfanger et al. 2001; Boissinot and Bergeron 2002). The investigators met with the nursing practice council and worked with the shared governance group of each nursing unit regarding the following: inappropriate holding of doses, and importance of obtaining levels at the exact time requested. In-services were organized to educate the nursing staff and clinical pharmacists assigned to the different nursing units worked with individual nurses as needed. In addition, the investigators went to different department meetings to explain the rationale of the project and to seek other physicians’ cooperation, including resident physicians.

Vancomycin dosing strategies utilized by the TDM were in accordance with the 2009 vancomycin consensus guidelines (Rybak et al. 2009). A Microsoft Excel^®^ spreadsheet was designed to calculate the maintenance dose to reach a specified target trough and for subsequent dose adjustments. The formula utilized was based on a one-compartment model and the details of the pharmacokinetic calculations are available in a Additional file 1 (Winter 2003). Steady state conditions were considered to be achieved when 4 half-lives (~94% to steady state) had elapsed, during which time the dose and dosing frequency remained the same, and the renal function was stable.

It is important to note that the CTRL group was prior to the release of the 2009 vancomycin dosing consensus guidelines, but high trough vancomycin dosing was already being utilized by many providers for infections in which MRSA was a concern due to the 2005 American Thoracic Society (ATS) guidelines on nosocomial pneumonia (American Thoracic Society 2005). During the CTRL period pharmacists were available to assist with vancomycin dosing, but this was not routinely documented in the chart. In addition, in the ICU, a critical care pharmacist was available during regular business hours, excluding weekends. Of note, the goal target trough was always stated explicitly in the pharmacist note for the TDM group, and for the CTRL group it was determined by chart review of physician notes.

The intervention began on hospital admission with the physician ordering vancomycin and the pharmacist calculating the appropriate initial dose after determining the optimal target trough in consultation with the physician. The pharmacist then placed the vancomycin order and arranged the next vancomycin trough level time. This data and any further vancomycin dose adjustments and follow-up trough levels were documented daily in the electronic medical record. Loading doses were generally given to critically ill patients with serious infections at the discretion of the pharmacist and physician.

### Data collection, study definitions

Data collected from patients’ medical records included demographics, culture data, pharmacokinetic parameters, and clinical outcomes. Site of infection was identified via physician notes. The Co-morbidity Index and Score of Charlson (CCI) was calculated for each patient at the time of initiation of vancomycin therapy with a Microsoft Excel^®^ spreadsheet that is shared electronically through BioMed Central (Hall et al. 2004). Immunosuppression was defined as patients receiving any systemic corticosteroids prior to or during treatment with vancomycin, a positive HIV antibody test result, chemotherapy within the past 45 days, neutropenia resulting from the administration of chemotherapy, and recipients of an organ transplant (renal, liver, heart, or bone marrow) (Hidayat et al. 2006).

### Monitoring data

The following monitoring data was collected: receipt of a loading dose, total vancomycin dosage (g), target trough (15–20 or 10–15 mg/L), value of trough if >20 mg/L, initial dose, total troughs drawn, and the number of mistimed troughs.

### Microbiological data

All positive bacterial cultures were recorded. If MRSA was cultured, the MIC (minimum inhibitory concentration) for vancomycin was documented. During the study periods, the laboratory determined the MIC for MRSA via VITEK^®^ 2 (Biomerieux), with isolates showing MICs 2–4 mg/L subjected to E-test confirmation.

### Nephrotoxicity analysis

As the TDM utilized more intensive vancomycin dosing practices, we had to enhance our safety monitoring for nephrotoxicity as this is the primary vancomycin dose-related toxicity of concern to clinicians (Lodise et al. 2009). Nephrotoxicity was defined and graded as acute kidney injury (AKI) via the RIFLE [Risk, Injury, Failure, Loss, ESRD (End Stage Renal Disease)] Criteria (Hoste et al. 2006; Minejima et al. 2011; Shen et al. 2011). The reported etiology of the acute kidney injury in the chart was extracted from provider notes (Clemens et al. 2011). In addition, whether or not vancomycin was implicated as the cause of the AKI by the clinician in the chart was also recorded (Clemens et al. 2011). We departed from the typical definition of vancomycin nephrotoxicity (Rybak et al. 2009) as we felt it is difficult to apply such a definition and to establish causality retrospectively. Similar approaches are being utilized in the literature with both RIFLE and AKIN (AKI Network) criteria (Minejima et al. 2011; Shen et al. 2011). In addition, concomitant nephrotoxins, and whether those with AKI had an initial vancomycin trough >20 mg/L were evaluated. Nephrotoxicity was not evaluated among subjects with end-stage renal disease requiring hemodialysis prior to initiation of vancomycin (Clemens et al. 2011).

### Outcome analysis

Primary outcome measures included time to target trough (days), time to clinical stability (defined below), all cause in-hospital mortality, and the inpatient length of stay (days). Other outcomes included vancomycin treatment failure, time to normalization of the WBC count (normal range = 4,500–10,000 cells/mL) in days, and inpatient lengths of vancomycin therapy (days). Time to clinical stability was defined as the return of vital signs to normal baseline values (heart rate <100 beats/min, systolic blood pressure >90 mmHg, respiratory rate <24 breaths/min, oxygen saturation >90%, and temperature <37.2°C) (Hidayat et al. 2006). Patients that met the definition of clinical stability at the time of admission were excluded from the time to clinical stability analyses; and patients that had a normal WBC at admission were excluded from the analyses of time to reach normal WBC. A parameter was considered to be stable if all measurements met the criteria for normality over a 24-h period (Halm et al. 1998).

There are a variety of definitions of clinical responses, clinical cure and clinical failure utilized in the literature (Kullar et al. 2011a; Hidayat et al. 2006; Stryjewski et al. 2007). However, there is no consensus on the definition of treatment failure in the literature (Sánchez 2009). For this study vancomycin treatment failure was defined as re-initiation of vancomycin therapy for any reason during the hospital stay after a full course of treatment or a switch to an alternate MRSA agent (daptomycin, linezolid, or tigecycline), but not including a true vancomycin allergy.

### Statistical analysis

Statistical analyses were conducted using SAS software v 9.2, Cary, NC, USA. A two-sided Fisher’s exact test was used to compare differences in proportions between the VPS and pre-intervention groups for categorical variables and two-sided Wilcoxon test was used for continuous variables. Kaplan–Meier (K–M) and Cox proportional hazard models were used to assess differences for duration variables. A multivariable logistic regression analysis was used to estimate odds ratios for mortality.

For the K–M and Cox proportional hazards analyses, time to clinical stability, time to reach a therapeutic trough, length of vancomycin treatment and time to white blood cell normalization were treated as right censored and set to the length of stay if the patient died or was discharged before reaching the endpoint. A stepwise approach using an inclusion criterion of p < 0.05 was done to develop multivariable Cox proportional hazard models. Analyses based on selected subsets of data used the same predictors as the overall model.

## Results

### Nephrotoxicity analysis

The characteristics of patients who developed AKI to determine TDM safety are summarized in Table 1. There were 340 patients meeting criteria for the nephrotoxicity analysis [TDM (n = 173); CTRL (n = 167)], and of those 43 (13%) developed AKI. AKI occurrence was similar between the CTRL (n = 20) and TDM (n = 23) groups (p = 0.7). In addition, of the 340 patients included in the safety analysis, 50 (15%) had initial vancomycin troughs >20 mg/L, and was similar between the CTRL (n = 26) and TDM (n = 24) groups (p = 0.76). Of note, most of the patients who developed AKI had underlying renal insufficiency, were admitted to the ICU, had sepsis or septic shock, and were on a number of concurrent nephrotoxins (Table 1). There were no significant differences in RIFLE scores between the CTRL and TDM groups. No patients in either group were assigned a RIFLE score of ESRD. Vancomycin was equally implicated by clinicians as a cause for nephrotoxicity in both the CTRL (n = 4), and TDM (n = 2) groups (4 vs. 2%, p = 0.3). The occurence of AKI with an initial vancomycin trough >20 mg/L was no different between groups (25 vs. 27%, p = 0.74). However, those in the TDM group had a significantly lower median initial vancomycin trough (22 vs 31, p = 0.05). The cases of AKI attributed solely to vancomycin were few, with 1 case in the TDM and 2 cases in the CTRL group (p = 1.0). The remaining cases in which vancomycin was implicated were considered multifactorial.

**Table 1:** Characteristics of patients who developed Acute Kidney Injury to determine Vancomycin Therapeutic Drug Monitoring Program (TDM) safety compared to the historical control (CTRL)

	TDM (n = 173)	CTRL (n = 167)	P-value
Patients with acute kidney injury, n (%)	23 (13.3)	20 (12)	0.715
Male, n (%)	15 (65)	16 (80)	0.32
Age, median (IQR)	65 (48,75)	66 (50,76)	0.11
Weight (kg), median (IQR)	84 (74,114)	85 (74,112)	0.26
Co-morbidity Score, median (IQR)	8 (4,9)	6 (3,10)	0.29
Co-morbidities			
Chronic kidney disease (Stage III and IV), n (%)	8 (34.8)	5 (25)	0.52
Central nervous system, n (%)	7 (30.4)	9 (45)	0.36
Cardiovascular, n (%)	18 (78)	18 (90)	0.42
Pulmonary, n (%)	8 (35)	6 (30)	1.0
Diabetes, n (%)	11 (48)	6 (30)	0.35
Gastrointestinal, n (%)	8 (35)	4 (20)	0.33
Malignancy, n (%)	8 (35)	8 (40)	0.76
Rheumatologic, n (%)	5 (22)	3 (15)	0.7
Immunosuppression, n (%)	3 (13)	2 (10)	1.0
Site of infection			
Respiratory, n (%)	12 (52)	13 (65)	0.54
Bacteremia, n (%)	14 (61)	12 (60)	1.0
Urinary Tract, n (%)	2 (9)	4 (20)	0.39
Skin and Soft Tissue, n (%)	0	2 (10)	0.21
Bone/Joint, n (%)	1 (4)	3 (15)	0.32
Central Nervous System, n (%)	0	0	
Intra-abdominal, n (%)	1 (4)	0	1.0
Sepsis/septic shock, n (%)	12 (52)	14 (70)	0.35
Culture result			
MRSA, n (%)	10 (43)	4 (20)	0.12
MSSA, n (%)	1 (4)	2 (10)	1.0
Coagulase Negative Staphylococci, n (%)	5 (22)	4 (25)	1.0
*S. pyogenes*, n (%)	0	1 (5)	1.0
*E. faecalis*, n (%)	4 (17)	3 (15)	1.0
Other gram positives, n (%)	4 (17)	5 (25)	1.0
Gram negatives, n (%)	9 (39)	11(55)	0.37
Cultures negative, n (%)	2 (9)	4 (20)	0.39
MRSA MIC (mg/L)			
2, n (%)	0	0	
Site of admission			
ICU, n (%)	16 (70)	15 (75)	0.74
Medical Floor, n (%)	3 (13)	5 (25)	0.45
Surgical Floor, n (%)	4 (17)	0	0.11
Concurrent antibiotics			
β-Lactam, n (%)	22 (96)	18 (90)	0.59
Piperacillin/tazobactam, n (%)	17 (74)	11(55)	0.22
Cefepime, n (%)	2 (9)	6 (30)	0.12
Carbapenem, n (%)	1 (4.3)	1 (5)	1.0
Other, n (%)	2 (9)	0	0.49
Fluoroquinolone, n (%)	13 (57)	9 (45)	0.55
Aminoglycoside, n (%)	1 (4.3)	2 (10)	0.590
Patients with initial VAN trough >20 mg/L, n (%)*	6 (25)	7 (27)	0.74
Trough value, median (IQR)	22 (21,23)	31 (25,51)	0.05
RIFLE criteria			
Risk, n (%)	7 (30.4)	3 (15)	0.294
Injury, n (%)	6 (26.1)	8 (40)	0.515
Failure, n (%)	8 (34.8)	8 (40)	0.761
Loss, n (%)	2 (8.7)	1 (5)	1.00
ESRD, n (%)	0	0	
Clinician identified etiology of AKI			
VAN implicated, n (%)	2 (8.7)	4 (19)	0.32
Etiology identified as multifactorial, n (%)*	1 (50)	2 (50)	1.0
VAN as the only cause, n (%)*	1 (50)	2 (50)	1.0
Acute interstitial nephritis from other antimicrobial, n (%)	6 (26)	2 (10)	0.250
Acute tubular necrosis, n (%)	14 (60.9)	14 (70)	0.75
Contrast nephropathy, n (%)	1 (4.3)	1 (5)	1.00
Other, n (%)	3 (13)	4 (20)	0.687
VAN implicated and initial trough >20 mg/L, n (%)	2 (100)	4 (100)	1.0
Concurrent nephrotoxins, n (%)			
Vasopressors, n (%)	6 (26.1)	11 (55)	0.07
Diuretics, n (%)	13 (56.5)	12 (60)	1.00
Amphotericin B, n (%)	0	1 (5)	0.465
Angiotensin-converting-enzyme inhibitor or Angiotensin II receptor blocker, n (%)	6 (26.1)	3 (15)	0.467

* Percentages based on the total cases in which vancomycin was implicated in each group.

### Patients in the clinical outcomes analysis

Clinical outcomes analysis was only conducted on patients with culture confirmed gram positive infections that were sensitive to vancomycin, and with normal renal function, which resulted in 195 exclusions leaving 145 patients (79 patients in the CTRL group and 66 from the TDM group) (Figure 1). The baseline characteristics of the patients in the CTRL and the TDM group were similar (Table 2). For the MRSA subset, there were 36 patients in the CTRL and 35 in the TDM groups. The baseline characteristics of the patients with MRSA infections in the CTRL and the TDM group were similar (Table 2).

**Table 2:** Baseline patient characteristics of all culture confirmed gram positive infections and the MRSA infection subset

	All Gram Positive Infections			MRSA Infections		P value
	TDM N = 66	CTRL N = 79	P value	TDM N = 36	CTRL N = 35	
Male, n (%)	60 (90)	71 (91)	1.0	29 (81)	33 (94)	0.15
Age, median (IQR)	61 (44,72)	60 (47,70)	0.66	61 (45,73)	60 (48,67)	0.43
Weight (kg), median (IQR)	88 (70,101)	89 (68,115)	0.62	89.8 (76,99.8)	80 (61,109)	0.74
Co-morbidity Score, median (IQR)	3.5 (0,6)	4 (0,5)	0.23	4 (0,6)	3 (0,4)	0.07
Co-morbidities						
Central nervous system, n (%)	21 (32)	29 (37)	0.60	10 (28)	17 (49)	0.45
Cardiovascular, n (%)	39 (59)	52 (66)	0.49	25 (69)	22 (63)	0.62
Pulmonary, n (%)	23 (35)	19 (24)	0.20	13 (36)	6 (17)	0.11
Diabetes, n (%)	20 (30)	31 (39)	0.30	15 (42)	13 (37)	0.81
Gastrointestinal, n (%)	20 (30)	17 (22)	0.26	14 (39)	8 (23)	0.2
Malignancy, n (%)	7 (11)	12 (15)	0.47	6 (17)	1 (3)	0.12
Rheumatologic, n (%)	6 (9)	9 (11)	0.79	4 (11)	3 (9)	1.0
Immunosuppression, n (%)	11 (17)	10 (13)	0.64	5 (14)	2 (6)	0.43
Site of infection						
Respiratory, n (%)	17 (26)	23 (29)	0.71	6 (17)	11 (31)	0.17
Hospital associated pneumonia/hospital acquired pneumonia, n (%)	7 (11)	10 (13)	0.8	6 (17)	5 (7)	1.0
Ventilator associated pneumonia, n (%)	1 (2)	3 (4)	0.6	0	1 (3)	1.0
Bacteremia^a^, n (%)	20 (30)	23 (29)	1.0	11 (31)	9 (26)	0.79
Endocarditis, n (%)	1 (2)	2 (3)	1.0	1 (3)	1 (3)	1.0
Catheter/device associated, n (%)	2 (3)	1 (1)	0.6	1 (3)	1 (3)	1.0
Urinary tract, n (%)	12 (19)	10 (13)	0.36	2 (6)	5 (14)	0.26
Skin and soft tissue, n (%)	40 (63)	38 (48)	0.10	25 (69)	20 (57)	0.33
Cellulitis/subcutaneous abscess, n (%)^b^	39 (59)	37 (47)	0.14	23 (64)	20 (57)	0.63
Necrotizing fasciitis, n (%)	1 (2)	1 (1)	1.0	1 (3)	1 (3)	1.0
Bone/joint, n (%)	8 (12.1)	13 (17)	0.49	4 (11)	5 (14)	1.0
Osteomyelitis, n (%)	6 (9)	5 (6)	0.76	6 (17)	2 (6)	0.26
Septic arthritis, n (%)	4 (6)	3 (4)	0.7	2 (6)	1 (3)	1.0
Central nervous system, n (%)	0	1 (1)	1.0	0 (0)	1 (3)	1.0
Intra-abdominal, n (%)	1 (2)	1 (1)	1.0	0 (0)	0 (0)	1.0
Sepsis and septic shock, n (%)	13 (20)	21 (27)	0.43	4 (11)	9 (26)	0.13
Culture result						
MRSA, n (%)	36 (55)	35 (44)	0.25	–	–	–
MSSA, n (%)	10 (15)	16 (20)	0.52	–	–	–
Coagulase negative Staphylococci, n (%)	12 (18)	15 (19)	1.0	–	–	–
*S. pyogenes*, n (%)	4 (6)	1 (1)	0.18	–	–	–
*E. faecalis*, n (%)	10 (15)	19 (24)	0.21	–	–	–
Other gram positives, n (%)	15 (23)	12 (15)	0.29	–	–	–
MRSA MIC (mg/L)						
2, n (%)	5 (14)	9 (11)	0.77	5 (13.5)	9 (11.4)	0.77
Site of admission						
ICU, n (%)	12 (18)	21 (27)	0.24	10 (28)	9 (26)	1.0
Medical Floor, n (%)	33 (50)	36 (46)	0.62	12 (33)	15 (43)	0.47
Surgical Floor, n (%)	21 (32)	22 (28)	0.72	14 (39)	11 (31)	0.62
Sepsis and Septic Shock, n (%)	13 (20)	21 (27)	0.43	4 (11)	9 (26)	0.13
Concurrent antibiotics						
β-Lactam, n (%)	44 (67)	53 (68)	1.0	28 (78)	23 (66)	0.3
Piperacillin/tazobactam, n (%)	30 (68)	34 (64)	0.82	19 (53)	17 (49)	0.81
Cefepime, n (%)	5 (12)	6 (11)	1.0	3 (8)	2 (6)	1.0
Carbapenem, n (%)	4 (9)	3 (6)	0.7	1 (3)	2 (6)	0.61
Other, n (%)	5 (12)	10 (19)	0.4	5 (14)	2 (6)	0.43
Fluoroquinolone, n (%)	16 (24)	21 (27)	0.85	5 (14)	9 (26)	0.56
Aminoglycoside, n (%)	6 (9)	2 (3)	0.14	2 (6)	2 (6)	1.0
Other, n (%)	16 (24)	15 (19)	0.54	7 (19)	9 (26)	0.58

^a^Majority of bacteremias were secondary from extravascular sources (pneumonia, skin/soft tissue infection, etc.).

^b^Includes surgical site infections.

### Dosing and monitoring data

**Figure 2:**
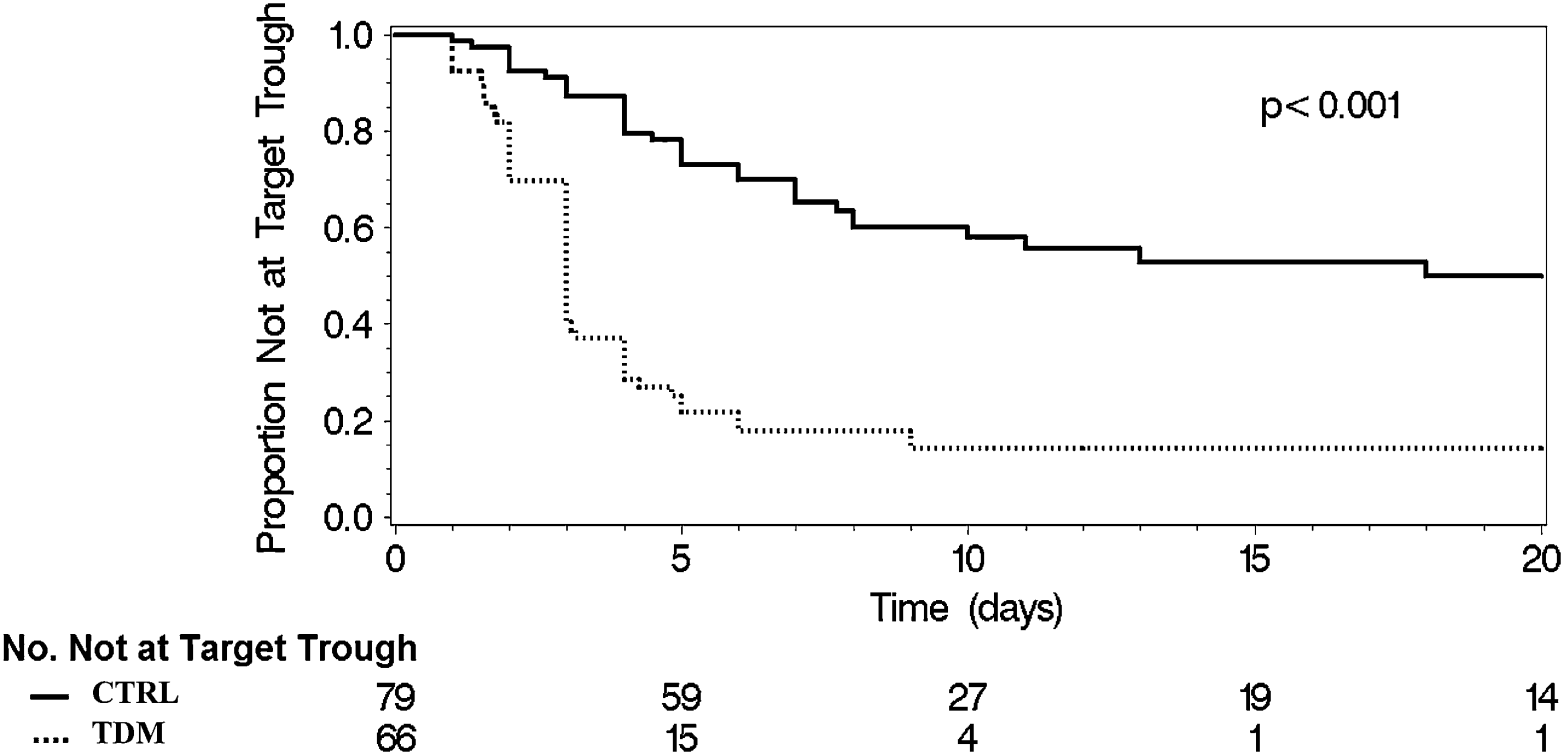
Unadjusted K-M plot demonstrating that those in the TDM group attained the target trough at an increased rate compared to the control group. *P* value generated via two sided Wilcoxon test.

More patients in the TDM group reached the initial target trough (Figure 2) 53 (80%) vs. 33 (42%) (P < 0.001), and the median time to initial target trough was shorter in the TDM group 3 (Interquartile Range (IQR), 2–3) vs. 5 (IQR, 2–7) days, p < 0.001). For the CTRL group there were 233 total troughs drawn with 47% being mistimed, and 185 drawn in the TDM with 32% being mistimed. There were significantly less total vancomycin troughs drawn, and less mistimed troughs drawn per patient in the TDM group (Table 3). Goal target troughs between the CTRL and TDM groups were similar: 15–20 μg/mL (33 vs. 52; p = 0.1), and 10–15 μg/mL (33 vs. 27; p = 0.1). The total vancomycin dose in grams per patient was similar between groups. Only 3 loading doses out of 66 patients (5%) were noted in the TDM group and none in the CTRL group. The initial dosing regimen in the TDM had significantly fewer doses of 1 g q12 h and had a wider variety, the most common being 1 g q8 h.

**Table 3:** Vancomycin dosing and monitoring parameters for those with culture-confirmed gram positive infections

	TDM (n = 66)	CTRL (n = 79)	P value
Reached Initial Target Trough, n (%)	53 (80)	33 (42)	<0.001
Time to Initial Target Trough (days), median (IQR)	3 (2,3)	5 (2,7)	<0.001
Initial target trough			
15–20 mg/L, n (%)	33 (50)	52 (66)	0.1
10–15 mg/L, n (%)	33 (50)	27 (34)	0.1
Loading dose, n (%)	3 (5)	0	0.54
Initial dose			
1.0 g q12hr, n (%)	28 (42)	63 (80)	<0.001
1.0 g q8hr, n (%)	12 (18)	1 (1)	0.001
Other, n (%)	26 (40)	14 (19)	0.005
No dose adjustment, n (%)	21 (32)	37 (48)	0.06
Number of troughs drawn per patient, median (IQR)	1 (1,4)	2 (1,4)	0.02
Mistimed troughs drawn per patient, median (IQR)	0 (0,2)	1 (0,2.5)	0.002
Total VAN Dose (g), median (IQR)	10 (7,20)	11 (7,21)	0.29

### Outcomes for all culture confirmed gram positive infections

Compared to the CTRL group, patients in the TDM group discharged from the hospital more rapidly, reached clinical stability faster, and had shorter courses of inpatient vancomycin treatment (Table 4). The CTRL and TDM group had similar all cause in-hospital mortality. Increased mortality was associated with age (OR 1.06; 95% CI 1.03–1.09; p < 0.001), ICU site of admission (OR 8.02; 95% CI 3.03–21.02; p < 0.001), respiratory site of infection (OR 4.08; 95% CI 1.66–10.06; p = 0.002), and bacteremia (OR 2.64; 95% CI 1.08–6.48; p = 0.034). Mortality was not associated with being in the CTRL or TDM groups. Vancomycin treatment failure was not significantly different between the TDM and CTRL groups [1 (1%) vs. 3 (4%); p = 0.62].

**Table 4:** Analysis of clinical outcomes for all culture confirmed gram positive infections and the MRSA infection subset

Outcome	Univariate			Multivariate	
	TDM	CTRL	P-value	TDM vs. CTRL	P-value
	Median (IQR)	Median (IQR)		Hazard Ratio (95% CI)	
All gram positive infections					
Inpatient length of stay (days)	7 (3,10)	14 (8,26)	0.03	1.41 (1.08–1.83)	0.01
Inpatient length of VAN Treatment (days)	4 (2,4)	7 (5,10)	<0.001	1.5 (1.15–1.95)	0.003
Time to clinical stability (days)	4 (3,5)	8 (3,7)	0.004	1.51 (1.08–2.11)	0.02
Time to normal WBC count (days)	4 (2,10)	6 (3,13)	0.14		
MRSA Subset					
Inpatient length of stay (days)	7 (4,20)	16 (7,38)	0.035	1.89 (1.08–3.3)	0.03
Inpatient length of VAN treatment (days)	5 (3,12)	8 (5,16)	0.034	2.52 (1.38–4.6)	0.003
Time to clinical stability (days)	4 (3,4)	6 (4,10)	0.013	2.69 (1.27–5.7)	0.01
Time to normal WBC count (days)	4 (2,5)	5 (2,6)	0.617		

### Outcomes for the MRSA Subset

For the MRSA subset analysis, compared to the CTRL group (n = 35), patients in the TDM group (n = 36) discharged from the hospital more rapidly, reached clinical stability faster, and had shorter inpatient courses of vancomycin treatment (Table 4). The CTRL and TDM group (2.9 vs. 2.8%, p = 1.0) had similar all cause in-hospital mortality. Vancomycin treatment failure was not significantly different between the TDM and CTRL groups (0 vs. 1 (3%); P = 0.5).

### Determination of optimal time to initial target trough

A separate Cox regression model examined only those patients who reached a target trough in a stepwise manner to determine the optimal time to initial target trough. Adjusted analysis revealed that attaining a target trough in less than 5 days versus greater than or equal to 5 days resulted in more rapid hospital discharge, patients reaching clinical stability faster, more rapid normalization of the WBC count, and shorter courses of inpatient vancomycin treatment (Table 5).

**Table 5:** Cox Proportional hazards regression model demonstrating that reaching the initial target trough in <5 days is associated with improved study outcome measures

Outcome	Hazard Ratio (95% CI)	P value
Inpatient length of stay^a^		
Time to target trough		
<5 vs. ≥5 days	2.52 (1.54–4.14)	<0.001
1–<2 vs. ≥5 days	1.97 (1.09–3.56)	0.024
2–<3 vs. ≥5 days	2.3 (1.35–3.92)	0.002
3–<4 vs. ≥5 days	2.13 (1.27–3.56)	0.004
Time to clinical stability^a^		
Time to target trough		
<5 vs. ≥5 days	2.13 (1.16–3.93)	0.015
1–<2 vs. ≥5 days	1.51 (0.75–3.04)	0.243
2–<3 vs. ≥5 days	1.09 (0.56–2.13)	0.791
3–<4 vs. ≥5 days	1.41 (0.76–2.62)	0.276
Inpatient length of VAN treatment^a^		
Time to target trough		
<5 days vs. ≥5 days	2.95 (1.8–4.82)	<0.001
1–<2 vs. ≥5 days	3.44 (1.86–6.35)	<0.001
2– < 3 vs. ≥5 days	2.18 (1.27–3.74)	0.005
3– < 4 vs. ≥5 days	4.67 (2.68–8.14)	<0.001
Time to normal white blood cell count^a^		
Time to target trough:		
<5 days vs. ≥5 days	2.08 (1.06–4.08)	0.034
1–<2 vs. ≥5 days	2.12 (0.86–5.21)	0.1
2–<3 vs. ≥5 days	1.26 (0.57–2.77)	0.565
3–<4 vs. ≥5 days	1.72 (0.84–3.53)	0.138

^a^Analysis restricted to those who reached target trough.

## Discussion

We were able to demonstrate that a pharmacist-run TDM program can safely and effectively decrease the median time to reach the initial vancomycin target troughs. Vancomycin TDM and target trough attainment in less than 5 days were associated with decreased inpatient lengths of stay, decreased inpatient lengths of vancomycin treatment, and decreased time to patient clinical stability. The TDM intervention was safe, as it was not associated increased rates of acute kidney injury, nor increased rates of vancomycin associated nephrotoxicity.

There are a number of studies similar to our patient population in the literature supporting vancomycin pharmacist managed therapeutic drug monitoring programs. Our study corroborates these studies in that we demonstrated that the TDM resulted in an increased percentage of patients reaching target trough, reduced the number of troughs per patient, and reduced the number of mistimed troughs. Morrison et al. (2012) noted that 41.3% of the vancomycin levels drawn in their institution were mistimed. This is consistent with the CTRL group in this study with 47% of troughs being mistimed; however the TDM intervention was able to significantly reduce mistimed troughs by 15%. Aubron et al. (2011) implemented a pharmacokinetic program in the ICU and was able to attain the target trough in 42% of patients, and the initial target trough in 40% of patients. In our study with our TDM intervention we were able to reach the initial target trough in 80% of patients; however our population was not restricted to only ICU patients, and vancomycin dosing is more challenging in the critical care setting. Similar to our study another study incorporated an educational intervention with a vancomycin dosing protocol, improving the proportion of patients who rapidly achieved optimal vancomycin exposures, and reduced the number of patients prescribed the traditional 1 g initial dose (Li et al. 2012). In our study, a 1 g q12hr dose was utilized significantly less in the TDM group and a wider variety of doses were utilized. This is an important point as it highlights the need for individualized dosing to achieve current guideline recommendations, rather than the traditional dogma of all patients receiving 1 g q12hr.

A number of authors have noted that a vancomycin TDM program was associated with a decreased incidence of vancomycin-induced nephrotoxicity (Bond and Raehl 2005; Iwamoto et al. 2003; Welty and Copa 1994). In our study, the occurrence of vancomycin associated nephrotoxicity was no different between the TDM and CTRL groups. However, the previously cited studies were not utilizing high trough dosing as in the current study. Given that the development of vancomycin-induced nephrotoxicity is dose related, the fact that the TDM utilized high trough dosing strategies and did not result in increased nephrotoxicity rates, highlights the safety of the intervention. The patients who developed acute kidney injury in this study were similar to other studies as many of them had baseline renal insufficiency, were ICU patients with changing hemodynamics, and were administered concomitant nephrotoxins (Hazlewood et al. 2010; Vandecasteele and De Vriese 2010). In addition, most patients in both groups were on concomitant piperacillin-tazobactam, which has recently been demonstrated to increase the incidence of nephrotoxicity in patients receiving vancomycin therapy (Burgess and Drew 2014). The rate of nephrotoxicity observed in this study (13% in the TDM and 12% in the CTRL groups) was similar to the literature range of 5–25% (Hazlewood et al. 2010; Iwamoto et al. 2003). The rate of nephrotoxicity in those with initial trough values >20 mg/L in the TDM (25%) and CTRL (27%) groups, was similar to another study that reported a rate of 33% (Lodise et al. 2009). Interestingly, of those who had AKI and initial trough values >20 mg/L, those in the TDM group had a significantly lower median vancomycin trough values, suggesting that individualized dosing may help to decrease the occurrence of exceedingly high trough values.

A unique aspect to our study was that the TDM significantly reduced the time interval to reaching target trough levels and this parameter was associated with improved outcomes. There is emerging evidence from the literature supporting vancomycin dosing recommendations for MRSA infections as put forth by the 2009 vancomycin consensus guidelines (Leu et al. 2012; Kullar et al. 2011b, 2012; Hall et al. 2012; Holmes et al. 2013). Kullar et al. (2011a) conducted a single-center retrospective analysis of 320 patients with documented MRSA bacteremia and found that 52.5% experienced vancomycin failure. By using regression analysis, they were able to demonstrate that patients with vancomycin area under the curve at 24 h (AUC_24h_) to MIC ratios <421 were found to have significantly higher rates of failure compared with patients with AUC_24hr_ to MIC ratios >421 (Kullar et al. 2011a). The same study group published a retrospective quasi-experimental study of 200 patients treated for confirmed, complicated MRSA bacteremia and compared patients prior to implementation of the vancomycin dosing guidelines to after (Kullar et al. 2012). Our results were similar to the most recent study by Kullar et al. (2012) in terms of reduced duration of vancomycin therapy, and not observing increased rates of nephrotoxicity. Most studies are now focusing on optimizing the AUC/MIC ratio and primarily in those with MRSA bacteremia, but few factor in the time interval to reach the goal AUC/MIC or target trough (Clemens et al. 2011; Hall et al. 2012; Holmes et al. 2013; Kullar et al. 2011a, 2012; Leu et al. 2012; Li et al. 2012; Nunn et al. 2011). Therefore, we explored whether reaching initial target troughs faster can impact outcomes and found that reaching an initial target trough in less than 5 days was associated with decreased length of hospital stay, decreased length of vancomycin treatment, decreased time to clinical stability, and decreased time to normalization of the WBC count. Holmes et al. (2013) recently demonstrated that that obtaining a vancomycin AUC/MIC > 373 was associated with decreased mortality, but only when accomplished within 4 days. Another study would seem to contradict our findings, and concluded that optimization of vancomycin pharmacokinetic indices to include time to target trough did not appear to correlate with clinical responses (Clemens et al. 2011). However, the time to target trough in that study was no less than 5 days in all comparison groups, as opposed to our TDM group, which was 3 days (Clemens et al. 2011).

We were surprised that despite our TDM intervention, only 5% of patients received an initial loading dose. This is likely because loading doses were not mandated for specific indications and generally given at the discretion of the pharmacist and/or physician. In a survey study of 163 hospitals in the Making Difference in Infectious Diseases Pharmacotherapy (MAD-ID) Research Network, 14% reported “never”, 43% reported “sometimes”, and 42% reported “always” utilizing a loading dose, indicating widespread underutilization of this practice (Davis et al. 2013). Reasons for this could include fears over administering large vancomycin doses or toxicity, delays in diagnosis and failure to recognize indications requiring a loading dose, lack of data suggesting improved outcomes, and years of habitually prescribing the “one-size-fits-all” 1 g every 12 h without a loading dose (Davis et al. 2013). We believe that all of these factors were responsible for the underutilization of loading doses in our study. One solution is to incorporate the emergency department into vancomycin TDM programs, and it may be possible to further reduce the time to target trough, and in turn, potentially improve patient outcomes. In support of this concept, a recent randomized study evaluated the percentage of troughs reaching therapeutic levels at 12, 24, and 36 h following an initial vancomycin loading dose of 30 mg/kg compared with 15 mg/kg in an emergency department (Rosini et al. 2015). In this study, there were a significantly greater proportion of patients reaching target trough levels of 15 mg/L among the patients who received a loading dose as compared with a traditional dose, and this trend continued at 24 h but was not statistically significant (Rosini et al. 2015). No statistically significant differences in nephrotoxicity or adverse events among were demonstrated, but no other clinical outcome measures were examined (Rosini et al. 2015).

Although we were unable to show an associated mortality benefit we demonstrated other important benefits to include expedited time to hospital discharge, and assisting patients in reaching clinical stability faster. Shorter durations of vancomycin therapy can decrease hospital costs, the risk of drug-induced toxicity and minimize the risks of nosocomial infection (Safdar and Maki 2002). Improved time to clinical stability may shorten lengths of stay in high acuity units, decrease overall hospital length of stay, and it has been demonstrated that once clinical stability has been achieved the risk of subsequent clinical deterioration may decrease to 1% or less even among the sickest patients (Halm et al. 1998). The lack of mortality benefit may be related to sample size, as after exclusions, the study was not adequately powered to detect a difference in mortality. In addition, the lack of mortality benefit may be due to the fact that we examined all-cause inpatient mortality rather than utilizing an alternate parameter such as infection-related or 30 day mortality. It has been estimated that the percentage of MRSA-related hospitalizations that resulted in death was approximately 6.2% in a large study (Klein et al. 2007). In our study, for all patients who were admitted with a culture confirmed MRSA infection, the mortality rate was lower at 2.8%. The lower mortality rate in our data may be related to the fact that the vast majority of MRSA isolates causing infection in our studies had vancomycin MICs of one or less as higher vancomycin MICs have been associated with increased mortality (Jacob and DiazGranados 2013). However, the MRSA isolates in our study with MICs of 2 mg/L or higher may have been under-represented as the Vitek 2 tends to under-call MICs of 2 mg/L in comparison to broth microdilution (Rybak et al. 2013).

Other limitations to our study include those inherent to the retrospective study design. Our study was at a single center, which has the advantage of eliminating potentially confounding site specific factors, but a potential disadvantage for generalization of the results to other facilities. In Hawaii, for example, Pacific Islanders have tended to have high rates of MRSA colonization and infection (CDC 2004). During the study period, the prevalence of MRSA infection and colonization ranged from 46 to 50% in our facility. This is similar to national prevalence data in the United States from 2010 (Jarvis et al. 2012). Also, we did not examine the AUC/MIC ratios in our patients. Although monitoring and targeting this parameter has recently been associated with improved outcomes in MRSA infections, it is not practical for clinical use at this time for most hospitals (Rybak et al. 2009). As this was a retrospective study, not enough vancomycin levels were drawn daily to calculate an accurate AUC/MIC in our opinion. In addition, to accurately estimate AUC_0–24hr_ with use of standard population parameters, a separate pharmacokinetic study would have to be conducted at our institution which was beyond the scope of the current study.

Future applications for vancomycin TDM programs include integration into electronic medical records (EMRs). Such an application could conserve pharmacist time and may result in further cost savings (Traugott et al. 2011). Another use would be in clinical trials comparing vancomycin therapy to new anti-MRSA antibiotics. For example, in the study claiming superiority for linezolid over vancomycin in hospital-acquired pneumonia, vancomycin may not have been dosed optimally as the vancomycin trough did not reach the recommended 15–20 mg/L range until day 9 of therapy (Wunderink et al. 2012). If target vancomycin troughs were achieved more rapidly, it may be more difficult for novel agents to demonstrate non-inferiority or superiority. Additionally, as AUC/MIC measurement methods become less cumbersome and in recent studies seem to correlate with outcomes more than trough values it will be important to examine if the time to reach the optimal AUC/MIC can further improve clinical outcomes.

## Conclusions

We suggest that interventions designed to decrease the time to reach initial target vancomycin troughs can improve clinical outcomes in gram positive infections, and in particular MRSA infections. Based on our data we recommend that the target trough be achieved before five days to optimize clinical outcomes. Further study of similar interventions would be of value in prospective, multi-center studies utilizing loading doses more frequently, and comparing intermittent versus continuous infusion regimens in conjunction with aggressive loading doses.

## Additional file

Additional file 1: Supplemental Methods: Vancomycin pharmacokinetic dosing guidelines.
