# Supplementary material for: Optimization of time to initial vancomycin target trough improves clinical outcomes
Source: Springerplus. 2015 Jul 19;4:364. doi: 10.1186/s40064-015-1146-9 (PMC4506278; doi:10.1186/s40064-015-1146-9)
Supplement: Additional file 1: — Supplemental Methods: Vancomycin pharmacokinetic dosing guidelines. [file 40064_2015_1146_MOESM1_ESM.docx]

**Supplemental Methods:**

VANCOMYCIN PHARMACOKINETIC DOSING GUIDELINES

I. **Required Patient Parameters**

All required patient parameters should be determined before calculating any dosage regimens.

A. Ideal Body Weight (IBW)

1. Male = 50 kg + 2.3 kg per inch over 5 feet (f.1)

2. Female = 45.5 kg + 2.3 kg per inch over 5 feet (f.2)

B. Weight used in Creatinine Clearance (CrCl) calculation

Adjusted body weight (ABW) should be used for creatinine clearance calculations if actual weight is >25% of IBW. If actual weight is <=25% of IBW, then use the actual weight (AW) for creatinine clearance calculation.

1. If AW <= 25% IBW, then use AW.

2. If AW is >25% higher than IBW, then ABW = IBW + (AW-IBW) x 0.4 (f.3)

C. Creatinine Clearance (CrCl)

1. CrCl (male) = (140 - age) x (AW or ABW) (f.4)

Scr x 72

2. CrCl (female) = CrCl (male) x 0.85 (f.5)

This provides an acceptable estimate of the patient's CrCl in ml/min except when Scr is changing rapidly.

1. Volume of Distribution (Vd)

Volume of distribution is 0.7 L/kg. The actual weight (AW) should be used when making this calculation since vancomycin distributes equally well to fat and lean body tissue.

Vd = AW x 0.7L/kg (f.6)

E. Vancomycin Clearance

1. Cl (male) = (140 - age) x ABW x 0.9 (f.7)

Scr x 72

2. Cl (female) = Cl (male) x 0.85 (f.8)

This provides an acceptable estimate of the patient's Cl in ml/min except when Scr is changing rapidly.

F. Vancomycin Rate Elimination Constant (hr-1)

K = Cl x 0.06 (f.9)

Vd

D. Half-life (hr)

t1/2 = ln 2 (f.10)

K

II. **Suggested Trough (Cpmin) Levels**

TABLE 1

| Types of Infection | Desired Vancomycin Trough Levels (mg/L) |
| --- | --- |
| Meningitis, osteomyelitis, pneumonia, sepsis, bacteremia, MIC=1 regardless of infection type | 15-20mg/L |
| Skin and soft tissue infection, urinary tract infection | 10-15mg/L |

III. **Loading Dose (LD)**

Once the above factors have been determined, the loading dose can be calculated as follows:

LD = (C_final_ - C_initial_) x Vd (f.11)

The usual loading dose is approximately 20-30 mg/kg for vancomycin (NTE 2000mg), which can be considered in patients with renal failure (since it takes a longer time to reach steady state) and seriously ill patients to rapidly reach the desired therapeutic range. For the above equation, C_initial_ is zero if it is the initial dose.

IV. **Maintenance Dose (MD) Calculations**

MD = (Css)(l-e^-kτ^)(Vd) (f.12)

(e^-kt^)

Css= concentration at steady state; τ= dosing interval, t= time from start of vancomycin infusion to when trough taken. Round dose to the nearest 250mg.

A good rule of thumb is to choose a dosing interval (τ) that is closest to the half-life. For example, if the half-life is 7 hours, choose 8 hours as the dosing interval. Typical dosing intervals are q8h, q12h, or q24h. Dosing interval below q8h (i.e. q6h) should not be used during the initial dosing unless patient specific population dictates it (i.e. after vancomycin level obtained).

Patients with rapidly changing serum creatinine should not be started on a standing maintenance dose. A loading dose should be given to reach desired therapeutic range rapidly, then vancomycin should be dosed per level (i.e. redose when vancomycin level falls within trough range).

V. **Monitoring Therapy**

A. For patients receiving vancomycin, the following baseline monitoring parameters should be recommended and followed at the recommended time intervals.

Baseline

height

weight

Scr

intake and output

Follow-up

weight every other day

Scr q3d; daily if renal function is changing

intake and output daily

1. Vancomycin trough level should be drawn when the patient is at steady state, which is usually before the 4^th^ dose. If the half-life and the dosing frequency are significantly different, use the half-life to determine when the steady state is achieved (4 half-lives ~93% to steady state).
2. Patients with rapidly changing renal function will never be at steady state. Thus, random levels are obtained in this scenario and patient is re-dosed when level reaches desired trough level.

VI. **Evaluating Serum Levels**

A. General Evaluation:

1. Was the level drawn at steady state? at the appropriate time? Were all doses given correctly?

2. Was the result of the level not expected? A comparison of predicted (population) versus actual values (patient specific) should be done.

B. Pharmacokinetic Evaluation of Trough Level:

1. Once a vancomycin trough level is obtained, patient specific pharmacokinetic parameters should be obtained. With only a trough level, only one independent pharmacokinetic parameter can be solved. Vd is less variable than Cl; therefore, the population Vd will have to be used during the revision. First calculate the patient specific K’ then the t’/12:

K’ = ln (((Dose/Vd)+C_p_trough)/C_p_trough) (f.13)

t_2_ - t_1_

Note that C_p_trough is the trough level. Please note that “((Dose/Vd)+C_p_trough)” and “C_p_trough” should be at least one half-life apart.

where t_2_-t_1_ is the difference in time (hrs) between “((Dose/Vd)+C_p_trough)” and “C_p_trough”

Half-Life:

t’1/2 = 0.693 (f.14)

K’

2. What if the trough level was drawn early? What if the trough level was drawn late? To extrapolate the actual trough…

a. C_p_actual trough = (C_p_trough-late) / e^(-kt)^ (f.15)

t = time between late level (C_p_trough-late) and actual trough (C_p_trough)

b. C_p_actual trough = (C_p_trough-early) * e^(-kt)^ (f.16)

t = time between the early level (C_p_trough-early) and actual trough (C_p_trough)

3. What is the patient specific Cl’?

a. Cl’ = K’ * Vd (f.17)

1. New Maintenance Dose

Now that the patient specific Cl’ and K’ have been calculated, and assuming that the patient specific Vd’ is very similar to the population Vd (i.e. Vd~Vd’), a new maintenance dose can be calculated. Remember to round dose to the nearest 250mg.

MD = (Css)(l-e^-k’τ^)(Vd) (f.18)

(e^-k’t^)

After the rounding, the actual trough concentration (Ctrough) can be calculated by rearranging f.11

Csstrough = [(D/Vd) (e^-k’t^) / (l-e^-k’τ^)] (f.19)

VII. **Pharmacokinetics Calculator**

All the equations cited above were programmed into an Excel calculator that is accessible via hospital intranet.
